# Postural responses to anterior and posterior perturbations applied to the upper trunk of standing human subjects

**DOI:** 10.1007/s00221-015-4442-2

**Published:** 2015-10-20

**Authors:** James G. Colebatch, Sendhil Govender, Danielle L. Dennis

**Affiliations:** Prince of Wales Clinical School and Neuroscience Research Australia, University of New South Wales, Randwick, Sydney, NSW 2052 Australia; Institute of Neurological Sciences, Prince of Wales Hospital, Randwick, Sydney, NSW 2031 Australia

**Keywords:** Posture, Postural reflexes, Stance

## Abstract

This study concerned the effects of brisk perturbations applied to the shoulders of standing subjects to displace them either forwards or backwards, our aim being to characterise the responses to these disturbances. Subjects stood on a force platform, and acceleration was measured at the level of C7, the sacrum and both tibial tuberosities. Surface EMG was measured from soleus (SOL), tibialis anterior (TA), the hamstrings (HS), quadriceps (QUAD), rectus abdominis (RA) and lumbar paraspinal (PS) muscles. Trials were recorded for each of four conditions: subjects’ eyes open (reference) or closed and on a firm (reference) or compliant surface. Observations were also made of voluntary postural reactions to a tap over the deltoid. Anterior perturbations (mean C7 acceleration 251.7 m*g*) evoked activity within the dorsal muscles (SOL, HS, PS) with a similar latency to voluntary responses to shoulder tapping. Responses to posterior perturbations (mean C7 acceleration −240.4 m*g*) were more complex beginning, on average, at shorter latency than voluntary activity (median TA 78.0 ms). There was activation of TA, QUAD and SOL associated with initial forward acceleration of the lower legs. The EMG responses consisted of an initial phasic discharge followed by a more prolonged one. These responses differ from the pattern of automatic postural responses that follow displacements at the level of the ankles, and it is unlikely that proprioceptive afferents excited by ankle movement had a role in the initial responses. Vision and surface properties had only minor effects. Perturbations of the upper trunk evoke stereotyped compensatory postural responses for each direction of perturbation. For posterior perturbations, EMG onset occurs earlier than for voluntary responses.

**Electronic supplementary material:**

The online version of this article (doi:10.1007/s00221-015-4442-2) contains supplementary material, which is available to authorized users.

## Introduction

Human upright stance must be maintained despite challenges from unexpected perturbations. Reflexes originating from the vestibular apparatus and proprioceptive reflexes from the legs both contribute to maintaining postural integrity (e.g. Horak et al. 1994). Larger postural perturbations require more complex responses, many “automatic”, and these have been explored using moving and rotating platforms. Such studies have shown the shortest latencies for calf muscles and longer latencies for more proximal muscles (Horak and Nashner 1986) as well as a role for central “set” in determining the size of responses (Horak et al. 1989). Normal subjects are also known to have less stability for AP displacements than for lateral ones (Horak et al. 2005).

We have recently investigated the properties of a reflex evoked by small truncal perturbations (Graus et al. 2013; Govender et al. 2015). Previous evidence existed for a reflex originating from axial structures, possibly muscle spindles within truncal muscles, and independent of any effects of ankle proprioceptors (Gurfinkel et al. 1981; Do et al. 1988; Bloem et al. 2000). Govender at al. (2015) found that small axial accelerations evoked short latency responses in soleus, consistent with a postural response, and that these were sufficient to restore the original posture. Given this evidence, it was natural to question whether these reflexes or the afferents responsible for them had a role in larger perturbations of the trunk, similar to those used clinically to assess postural stability in Parkinson’s disease (e.g. Hunt and Sethi 2006). It was not clear to what extent brief perturbations applied to the trunk (at the shoulders), similar to a shove or bump when standing, would show properties like those reported for moving platform displacements (similar to a slip), given that muscle afferent activation and stretch reflexes in particular would be much smaller for calf muscles following a truncal perturbation. We have therefore investigated the patterns of muscular activation, accelerations and the dependence upon vision and surface type for normal volunteers in response to brief perturbations applied to the shoulders both anteriorly and posteriorly.

## Methods

### Subjects

A total of fourteen healthy volunteers (mean age 26 ± 9 years, five females and nine males) with no history of dizziness, vertigo, inner ear pathology or neurological illness were recruited from the staff and students at the University of New South Wales and Prince of Wales Hospital, Sydney. Ten subjects were tested for main experiment (mean age 27 ± 9 years). Eight subjects, four from the main experiment, performed recordings to investigate voluntary postural reactions (mean age 24 ± 4 years). Written informed consent, in accordance with the Declaration of Helsinki, was obtained from all subjects prior to the study commencing. The study was approved by the local ethics committee.

### Instrumentation and data acquisition

**Fig. 1:**
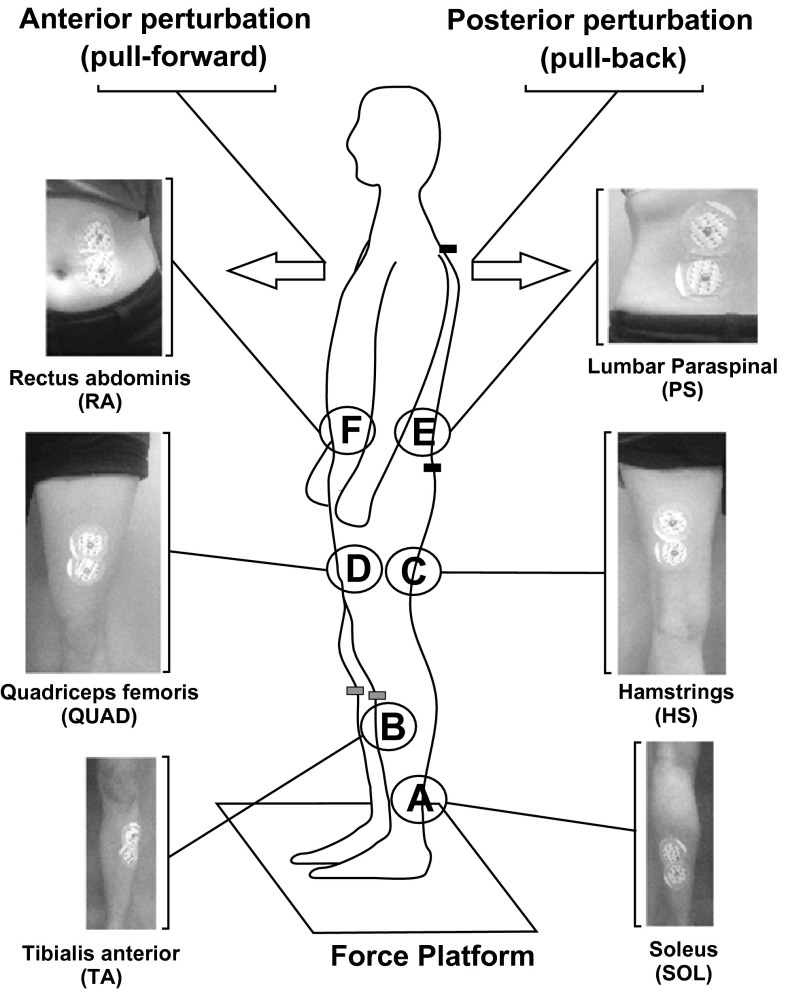
Recording arrangements. Subjects were asked to correct for postural disturbances (anterior or posterior perturbations) while standing upright on the force platform. EMG was recorded over the left side from the soleus (SOL: *A*) and tibialis anterior (TA: *B*), hamstrings (HS: *C*), quadriceps (QUAD: *D*), lumbar paraspinal (PS: *E*) and rectus abdominis muscles (RA: *F*) with accelerometers positioned over C7, the sacrum (shown in *black*) and the tibial tuberosities (shown in *grey*). Perturbations were applied at the level of the shoulders

Changes in centre of pressure (CoP) in the anterior–posterior (AP) and mediolateral (ML) axes were recorded using a force platform (model 9286 A, Kistler Instrumente, Winterthur, Switzerland). Pilot data from a larger pool of healthy subjects (*n* = 16) showed no significant difference in EMG latency or amplitude between the right and left soleus and tibialis anterior muscles. Thus, the main experiment consisted of unilateral EMG recordings from the left side over the tibialis anterior (TA), soleus (SOL), hamstrings (HS), quadriceps (QUAD), lumbar paraspinal (PS) and rectus abdominis (RA) muscles, with accelerometers placed over C7, the sacrum and bilaterally over the tibial tuberosities. Anterior displacements and accelerations were defined as positive. For SOL, active electrodes were placed 1–2 cm distal to the gastrocnemius musculotendinous junction. For TA, active electrodes were placed on the skin over the muscle belly, 1–2 cm lateral to the anterior border of the tibia. Active electrodes over the HS and QUAD muscles were positioned at the level of the midpoint between the ischial tuberosity and the popliteal fossa, 3–4 cm medial to the midline of the posterior thigh muscles and in the midline over the rectus femoris. For PS, the active electrodes were over *erector spinae* lateral to the middle of the lumbar spine and at the same level as the RA active electrodes which were 4–5 cm superior to the umbilicus and just medial to linea alba. For all muscle recording sites, the reference electrode was positioned 4–6 cm below the active recording electrode (Fig. 1).

EMG was amplified (1000×, D360 amplifier, Digitimer Co, Welwyn Garden City, UK), band-pass-filtered (8 Hz–1.6 kHz) and sampled using a Power1401 (Cambridge Electronic Design, Cambridge, UK). Rectified EMG data were constructed offline. Linear accelerometers were used (model 751–100, Endevco, California, USA), and the heights of the accelerometers above the force platform were measured. For the main set of recordings, accelerometers were placed over the tibial tuberosities bilaterally. Weight was measured using the output of the force platform prior to the perturbation. For the investigation of voluntary postural corrections, a tendon hammer fitted with a sensitive mechanical trigger was used (Nicolet Biomedical, WI, USA). Data were sampled at 4 kHz for 5 s (1.5 s pre-stimulus) and recorded using Signal software (version 3.13, Cambridge Electronic Design, Cambridge, UK).

### Experimental procedure

**Fig. 2:**
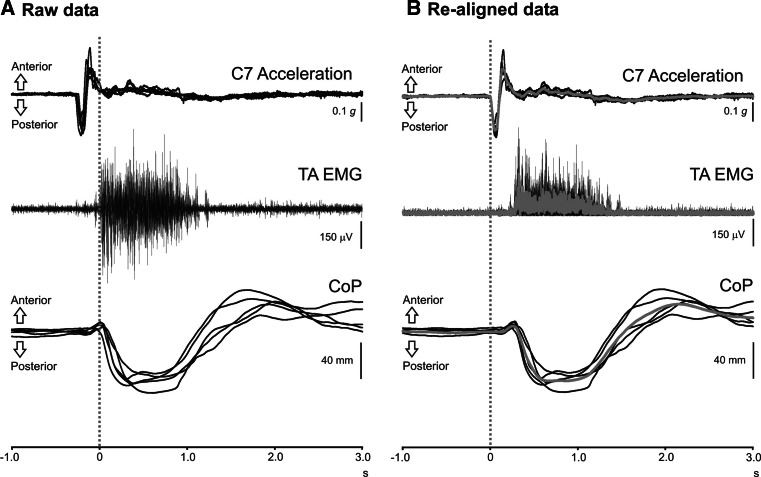
Realignment of raw data and averaging. Data shown from a single subject following posterior perturbations on a rigid surface during eyes open, triggered by the experimenter pressing a footswitch. The *left column* shows the five individual trials superimposed for C7 accelerations, unrectified EMG from tibialis anterior and CoP displacements (**a**). The *right column* shows each individual trial realigned to the onset of C7 accelerations and with EMG signals rectified (**b**). Averaged data for the realigned traces are shown in* grey* and were measured for data analysis. *Note* that the consistency of the footswitch trigger was good but that it systematically underestimated the true onset of the perturbation as shown by the C7 accelerometer

For the main experiment, ten subjects stood barefoot on the force platform while maintaining quiet upright stance and had their arms along the sides of their bodies. Subjects were instructed to attempt to correct their posture without moving their feet, following external perturbations performed by an experimenter (SG) standing either behind or in front of the subject. Perturbations were carried out by holding the subject at the shoulders and briefly pulling the torso in the anterior (pull-forward) or posterior (pull-back) direction. The experimenter pressed on a foot switch to trigger the averager at approximately the same time as the applied perturbation. EMG, accelerometry and CoP data were recorded simultaneously for all experiments, and the order of conditions presented was randomised. The output of the C7 accelerometer was immediately available after each trial, and a target of a nominal peak of 200 m*g* was used. The protocol consisted of eight separate sets of recordings. Subjects had four perturbations in both the anterior and posterior directions, being tested with either eyes open or eyes closed and while standing on either a rigid or compliant surface. The eyes open, rigid surface was taken to be the reference condition. For each set of recordings, five individual trials were recorded and averaged following offline manual realignment to the onset of C7 acceleration, which was defined as time 0 (Fig. 2). Postural reactions for the first presentation of each perturbation type were not measured as these have been shown produce greater amplitude responses than subsequent perturbations (Keshner et al. 1987; Oude Nijhuis et al. 2009). Perturbations sets were delivered randomly in order to avoid habituation and anticipation.

In eight subjects, recordings were performed to investigate voluntary postural corrections. Subjects were instructed to displace their posture as quickly as possible, by actively disturbing their natural stance (by leaning anteriorly or posteriorly) following tendon hammer taps to the right deltoid. The same stance conditions were used as for the main experiment.

### Post collection analysis

For each recording, the trace was manually realigned to the onset of C7 acceleration (time 0) and trimmed to include 1 s prior and 3 s post-onset. Then, the data from the five individual trials were averaged offline after full-wave rectification of EMG signals, all of which was done using MATLAB software (version 6.5.1, Mathworks Inc., Massachusetts, USA). Using this average, acceleration onsets and peaks and CoP onset and peak locations were selected manually and measured. The analysis program automatically measured baseline mean rectified EMG as well as activity in a series of time blocks following the onset of C7 acceleration (0–0.2, 0.2–0.5, 0.5–0.75, 0.75–1 then 0.5-s intervals to 3 s) and also measured the time for the CoP to return to half the distance between the peak and onset locations (‘mid-return time’). EMG onset latencies reflect median [range] values in tables and were determined from rectified EMG using a cumulative sum technique requiring 3 SD deviation from the preceding mean activity levels. For comparison of the different conditions tested, separate ANOVAs were performed for anterior and posterior perturbations, using surface type (rigid or compliant) and vision (eyes closed or eyes opened) as factors. These were done for acceleration, EMG latency and CoP data. For EMG amplitude, we used surface, vision and time interval as factors. To compare latencies for voluntary movements with those for perturbations, ANOVAs using task (voluntary lean or external perturbation), surface type and vision as factors were used and separate analyses were carried out for the anterior lean/posterior perturbation and posterior lean/anterior perturbation data sets. Statistical analysis was carried out using SPSS software (version 22.0, IBM Inc. Chicago, USA). Acceleration, EMG amplitude and CoP data are reported as mean ± SD in the text. Grand-averaged results were used for measuring the duration of the initial phasic EMG activation.

## Results

### Findings for the reference condition

#### Anterior perturbations

**Fig. 3:**
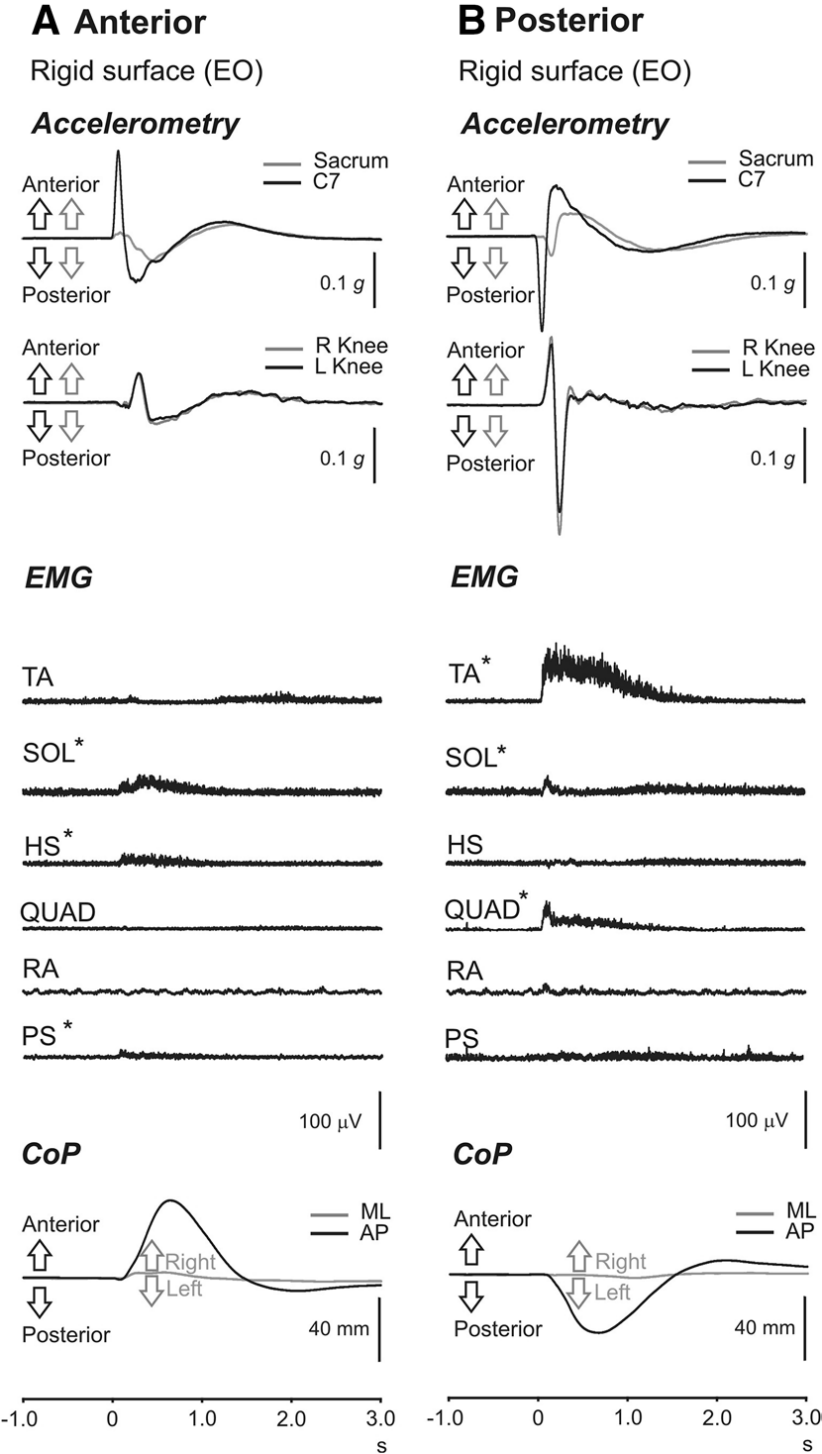
Anterior and posterior perturbations. Grand means of accelerometry, EMG and CoP recordings following anterior perturbations (**a**; *left column*) and posterior perturbations (**b**: *right column*) on a rigid surface during eyes open. Anterior perturbations showed an initial period with phasic bursts in soleus (SOL), hamstrings (HS) and paraspinal (PS) and prolonged contractions in SOL and HS. Posterior perturbations showed an initial phasic burst in quadriceps (QUAD) associated with cocontraction in TA and SOL, followed by prolonged activation in TA and QUAD. EMG responses were generally small for the HS, rectus abdominis (RA) and PS muscles. Upward deflections in accelerometry traces reflect anterior displacement in the anterior–posterior (AP) plane and rightward displacement in the mediolateral (ML) plane; *asterisk* denotes significant changes in rectified EMG compared to baseline levels

Accelerometers placed at C7, sacrum and the tibiae had mean heights of 146 ± 8 cm (range 132–159 cm), 100 ± 7 and 40 ± 2 cm, respectively, and the mean weight of subjects tested was 75 ± 19 kg (range 54–111 kg). Mean subject height was 170 ± 9 cm (range 156–183 cm). Grand means of acceleration, EMG and CoP traces following anterior perturbations on a rigid surface during eyes open are shown in Fig. 3a and overall values in Table 1. Anterior perturbations produced larger mean peak accelerations at C7 than for the sacrum. Mean peak latencies for C7 accelerations were earlier than for sacral accelerations. Tibial accelerations for the anterior perturbation showed a small initial posterior acceleration followed by a larger anterior one. Mean tibial accelerations were 72.5 and 61.4 m*g* for the right and left sides, respectively, and occurred at mean latencies of 279.3 and 279.0 ms. The magnitude and latency of the tibial accelerations were not significantly different between the sides (*P* > 0.05). The peak CoP displacement ranged from 44.7 to 80.4 mm with peak latencies between 534.3 to 762.3 ms. Neither height nor weight showed any correlation with peak CoP displacement for anterior perturbations (*r* = 0.079 and −0.168, *P* > 0.05).

**Table 1:** Acceleration, CoP and EMG values for anterior perturbation conditions

Acceleration	Rigid surface				Compliant surface			
	EO		EC		EO		EC	
	Peak amp. (m*g*)	Peak lat. (ms)	Peak amp. (m*g*)	Peak lat. (ms)	Peak amp. (m*g*)	Peak lat. (ms)	Peak amp. (m*g*)	Peak lat. (ms)
C7	251.7 (58.4)	74.1 (7.0)	224.3 (26.6)	72.8 (9.2)	240.6 (30.8)	67.8 (8.7)	215.1 (31.3)	70.1 (10.5)
Sacrum	45.5 (16.4)	131.5 (22.5)	47.6 (36.4)	124.2 (25.9)	49.8 (35.8)	130.0 (38.6)	38.0 (23.8)	113.5 (35.9)
Tibial	66.9 (30.8)	279.1 (35.6)	49.0 (22.0)	262.2 (54.6)	77.4 (39.8)	275.0 (28.3)	52.4 (29.5)	251.3 (58.4)

CoP	Displacement (mm)	Latency (ms)	Displacement (mm)	Latency (ms)	Displacement (mm)	Latency (ms)	Displacement (mm)	Latency (ms)
AP	60.1 (15.1)	656.9 (77.5)	48.8 (9.7)	708.8 (104.7)	53.9 (9.6)	662.7 (68.9)	46.5 (8.2)	614.3 (117.9)
Onset	–	99.2 (15.5)	–	95.1 (11.6)	–	103.4 (10.1)	–	102.7 (10.3)
Mid-return	–	1116.7 (171)	–	1226.2 (279.5)	–	1170.8 (145.9)	–	1262.0 (253.2)

EMG	Median lat. (ms)	Median lat. (ms)	Median lat. (ms)	Median lat. (ms)
TA	151.5 [101.5–268.0]	128.0 [80.8–249.0]	219.3 [108.0–302.3]	135.3 [91.3–216.5]
SOL	116.5 [103.3–213.3]	102.1 [80.8–173.8]	106.3 [75.0–248.5]	95.5 [70.5–136.8]
HS	117.8 [93.5–296.8]	115.0 [88.0–290.3]	105.5 [84.8–158.3]	98.6 [84.3–281.8]
PS	94.0 [77.0–209.8]	109.3 [75.8–208.3]	114.8 [75.3–192.0]	113.0 [67.0–211.3]

Values are given as mean (SD) for acceleration and CoP. Onset EMG latencies are given as median [range]. Tibial accelerations reflect the mean of the left and right sides. Positive and negative values (for acceleration and CoP measurements) indicate anterior and posterior directions, respectively

*EC* eyes closed, *EO* eyes open, *Amp.* amplitude, *Lat.* latency

**Fig. 4:**
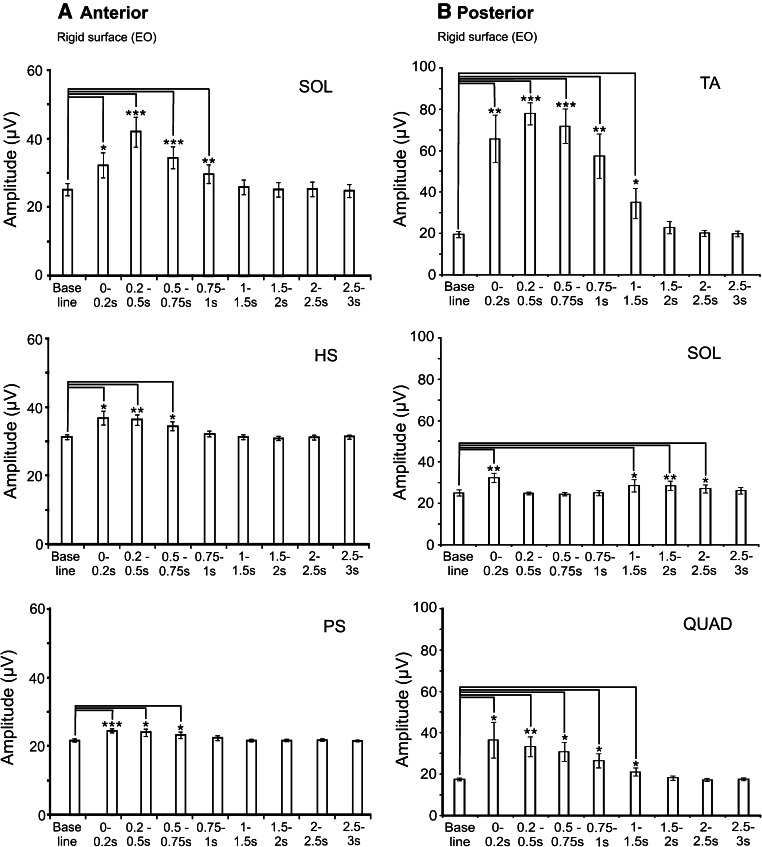
Rectified EMG levels during anterior and posterior perturbations. Muscle groups which demonstrated significant changes in mean rectified EMG levels from baseline are shown for anterior (*left column*) and posterior (*right column*) perturbations. Anterior perturbations showed significant increases from baseline for soleus (SOL), hamstrings (HS) and paraspinal (PS) muscles. The greatest modulation in amplitude for posterior perturbations was observed for tibialis anterior (TA), whereas a similar but less marked effect was seen for quadriceps (QUAD). SOL showed an initial increase in EMG amplitude from baseline to 0.2 s with a subsequent decrease over the 0.2- to 1-s time interval, followed by a later increase from 1 to 2.5 s

Anterior perturbations evoked a short initial phasic discharge in SOL, HS and PS, followed by more sustained activity (Fig. 3a, Supplementary Figure). ANOVA of rectified EMG levels from baseline to 3 s post-perturbation showed significant changes in activation for SOL, HS and PS muscles (Fig. 4a, *P* ≤ 0.003), while there were no significant changes for TA, QUAD and RA (*P* > 0.05). The greatest change in rectified EMG levels was observed for SOL which showed a significant increase in mean rectified levels from baseline to 1 s post-C7 onset. The largest mean rectified value for SOL was 41.8 ± 13.9 µV (baseline 25.0 ± 5.6 µV) which occurred at 200–500 ms post-perturbation. Similarly, the largest mean rectified levels for HS and PS occurred over the initial 200 ms post-perturbation and were 36.8 ± 6.5 and 24.6 ± 2.1 µV, respectively. Corresponding baseline EMG levels were 31.3 ± 2.2 µV (HS) and 21.7 ± 1.3 µV (PS). The EMG onset latency was shortest for PS but there was no significant difference between the TA, SOL, HS and PS latencies.

#### Posterior perturbations

Posterior perturbations produced peak C7 accelerations similar in magnitude to anterior perturbations but oppositely directed (Fig. 3b, Table 2). Tibial accelerations showed an initial anterior acceleration of the knees, in the opposite direction to that of the C7 and sacral accelerations. Mean tibial accelerations for the reference condition were 163.6 and 174.5 m*g* for the right and left sides, respectively, and occurred at mean latencies of 155.9 and 157.1 ms. The magnitude and latency of the tibial accelerations were not significantly different between sides (*P* > 0.05). Peak accelerations for C7 occurred earlier than peak sacral and tibial accelerations (*P* ≪ 0.001), but sacral acceleration latencies were not significantly different from tibial latencies (*P* = 0.094). CoP measurements showed displacements between −31.6 to −71.0 mm which occurred at latencies of 426.8 to 909.0 ms post-onset. Peak CoP displacement did not correlate with either height or subject weight (*r* = −0.081 and −0.210, *P* > 0.05).

**Table 2:** Acceleration, CoP and EMG values for posterior perturbation conditions

Acceleration	Rigid surface				Compliant surface			
	EO		EC		EO		EC	
	Peak amp. (m*g*)	Peak lat. (ms)	Peak amp. (m*g*)	Peak lat. (ms)	Peak amp. (m*g*)	Peak lat. (ms)	Peak amp. (m*g*)	Peak lat. (ms)
C7	−240.4 (45.4)	69.7 (12.3)	−231.2 (42.7)	69.9 (13.6)	−231.1 (26.0)	70.9 (9.7)	−236.3 (38.8)	71.1 (11.1)
Sacrum	−75.2 (33.8)	140.2 (21.3)	−67.8 (24.6)	139.9 (15.6)	−70.1 (30.8)	132.4 (25.9)	−67.8 (31.8)	133.8 (18.6)
Tibial	169.1 (131.4)	156.5 (28.5)	152.7 (104.5)	166.2 (17.3)	141.1 (120.1)	153.9 (19.8)	127.5 (88.7)	150.2 (30.2)

CoP	Displacement (mm)	Latency (ms)	Displacement (mm)	Latency (ms)	Displacement (mm)	Latency (ms)	Displacement (mm)	Latency (ms)
AP	−50.5 (14.6)	681.0 (139.0)	−52.4 (18.1)	654.1 (156.1)	−49.0 (14.9)	584.0 (135.0)	−52.3 (13.3)	537.9 (132.7)
Onset	–	90.7 (23.2)	–	99.4 (23.5)	–	95.4 (19.1)	–	97.8 (31.2)
Mid-return	–	1236.3 (228.6)	–	1287.7 (337.5)	–	1104.7 (200.0)	–	1194.9 (241.6)

EMG	Median lat. (ms)	Median lat. (ms)	Median lat. (ms)	Median lat. (ms)
TA	78.0 [63.5–132.8]	74.0 [53.5–106.8]	75.8 [48.0–121.0]	73.1 [56.3–254.5]
SOL	107.8 [82.5–158.8]	119.5 [80.5–192.5]	117.8 [64.3–259.3]	118.1 [65.5–253.0]
QUAD	89.8 [50.0–142.3]	83.8 [54.8–128.8]	73.0 [60.5–141.8]	82.3 [46.2–141.0]

Values are given as mean (SD) for acceleration and CoP. Onset EMG latencies are given as median [range]. Tibial accelerations reflect the mean of the left and right sides. Positive and negative values (for acceleration and CoP measurements) indicate anterior and posterior directions, respectively

*EC* eyes closed, *EO* eyes open, *Amp.* amplitude, *Lat.* latency

Grand-averaged EMG responses showed strong activation in the TA and QUAD muscles with an initial rapid activation associated with a period of cocontraction (73–141 ms post-perturbation) of the SOL muscles followed by a phase of prolonged EMG responses (Fig. 3b, Supplementary Figure). Rectified EMG measurements showed significant changes for TA, SOL and QUAD (Fig. 4b; *P* ≤ 0.002). There was no significant change in mean rectified EMG for HS, RA or PS muscles (*P* > 0.05). TA showed the most significant and largest increase in activity (78.0 ± 16.7 µV at 200–500 ms post-perturbation, baseline 19.7 ± 4.5 µV) and was significantly increased compared to baseline from the first period until 1.5 s post-perturbation. Likewise, QUAD was significantly activated over the same period with the largest mean rectified level at 0–200 ms post-perturbation (36.4 ± 27.1 µV, baseline 17.5 ± 2.2 µV). SOL was increased only for 0–200 ms post-perturbation (32.2 ± 7.1 µV, baseline 25.1 ± 4.9 µV), consistent with the period of cocontraction and became significantly increased again only after 1 s. There was no difference in EMG onset latencies between TA and QUAD muscles, but both were earlier than SOL onset latencies (*P* = 0.018 and 0.040, Table 2).

#### Anterior versus posterior perturbations

There was no difference in the magnitude or latency of C7 accelerations evoked by anterior and posterior perturbations (*P* > 0.05). The magnitude of sacral accelerations was larger for posterior perturbations (*P* = 0.027), but peak sacral latencies were not different (*P* > 0.05). Tibial accelerations for posterior perturbations were also generally larger (*P* = 0.066) and occurred significantly earlier (*P* ≪ 0.001) than for anterior perturbations. Peak CoP amplitudes, peak CoP latencies and CoP mid-return latencies did not differ significantly between the two directions (*P* > 0.05). There was no significant difference in baseline rectified EMG levels for any muscle group (*P* > 0.05) for the two directions.

### Voluntary reactions

**Fig. 5:**
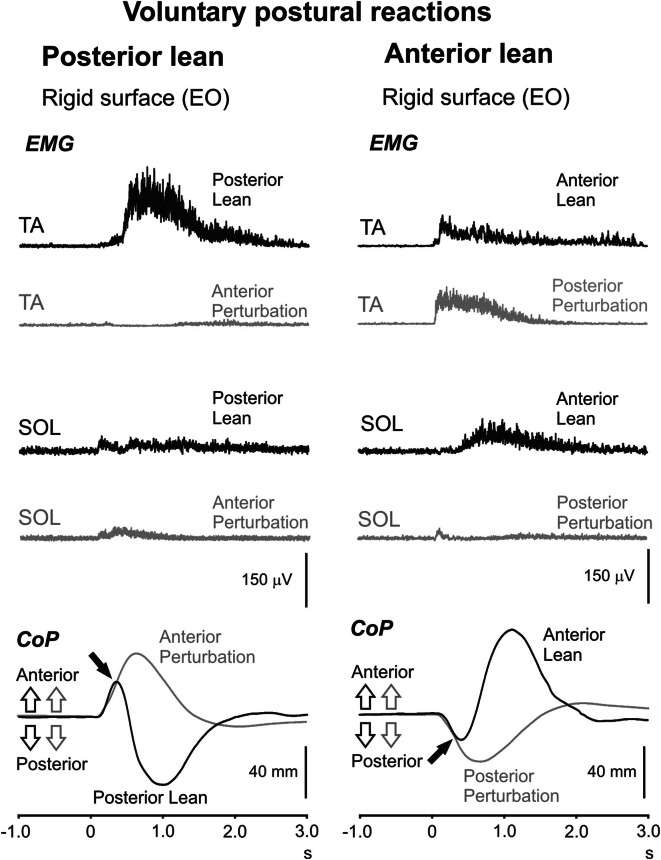
Voluntary postural reactions. EMG and CoP recordings following active posterior and anterior lean (*n* = 8) triggered by a tap to the shoulder. For comparison, responses to posterior and anterior perturbations are shown in* grey*. For posterior lean, SOL is active initially (the later TA activity acts to restore the initial posture), while the opposite applies to anterior lean. Initial cocontraction was not evident for either direction of voluntary movement. *Note* similar initial displacements for the CoP (indicated by the* black arrows*)

Voluntary posterior and anterior lean produced strong activations of both SOL and TA as well as brisk displacements of the CoP (Fig. 5). EMG profiles were notable for the absence of any initial cocontraction, in contrast to the imposed perturbations. The onset of CoP displacements occurred on average just over 120 ms after the shoulder tap and was longer than for the imposed perturbations (Table 3).

**Table 3:** Acceleration, CoP and EMG values for voluntary anterior and posterior lean for the reference condition

Acceleration	Anterior lean		Posterior lean	
	Peak amp. (m*g*)	Peak lat. (ms)	Peak amp. (m*g*)	Peak lat. (ms)
C7	28.5 (12.7)	275.3 (78.4)	−30.9 (13.9)	270.9 (59.8)
Sacrum	20.3 (18.0)	355.3 (71.1)	−35.7 (28.6)	285.2 (83.0)

CoP	Displacement (mm)	Latency (ms)	Displacement (mm)	Latency (ms)
AP	70.1 (37.6)	1149.0 (256.1)	−55.3 (22.3)	1041.3 (103.5)
Onset	–	124.8 (10.7)	–	121.8 (15.7)
Mid-return	–	1530.5 (275.9)	–	1517.4 (255.6)

EMG	Median lat. (ms)	Median lat. (ms)
TA	134.6 [106.9–197.9]	151.1 [108.6–161.8]
SOL	174.9 [158.9–248.0]	126.1 [104.8–160.8]

Values are shown for the reference condition (rigid surface with eyes open). *Amp.* amplitude, *Lat.* latency. Values are given as mean (SD) for acceleration and CoP. Onset EMG latencies are given as median [range]. Positive and negative values (for acceleration and CoP measurements) indicate anterior and posterior directions, respectively

Posterior lean and anterior perturbations demonstrated similar SOL mean onset latencies (139.1 and 133.5 ms, *P* > 0.05). For EMG amplitude, SOL responses for posterior lean and anterior perturbations were mostly similar over the recording period (interaction between task and time interval; *P* = 0.049) with only the initial 200 ms showing a larger amplitude for posterior lean (48.5 ± 17.1 vs 32.9 ± 14.1 µV, *P* = 0.017). In contrast, both onset latency and EMG amplitude showed significant differences between the anterior lean and posterior perturbation tasks. TA onset latencies were significantly earlier for posterior perturbations than for anterior lean (mean 88.1 vs 124.3 ms, main effect of task, *P* ≪ 0.001). TA amplitudes were also significantly larger for posterior perturbations than for anterior lean (main effect of task; *P* = 0.007), and these differences were dependent on the interval post-onset (interaction between task and time interval; *P* ≪ 0.001). Posterior perturbations produced larger TA responses than anterior lean from 200 ms to 1 s post-onset (Fig. 5: 200–500 ms: *P* ≤ 0.010).

### Effects of vision and surface type

For anterior perturbations, C7 accelerations were slightly larger during eyes open (mean EC −219.7 m*g*, mean EO −246.2 m*g*; *P* = 0.025) and peaked slightly earlier for the compliant surface (mean rigid 73.5 ms, mean compliant 69.0 ms, *P* = 0.022). There was no effect of the different conditions on the magnitude or latency of sacral accelerations (Table 1, *P* > 0.05). Tibial accelerations were larger during eyes open (mean EC −72.2 m*g*, mean EO −50.7 m*g*; *P* = 0.002), but latencies were not different across conditions (*P* > 0.05). For posterior perturbations, there were no significant effects of surface type and vision conditions for the magnitude or latency of C7, sacral or tibial accelerations (Table 2, *P* > 0.05).

The onset of CoP displacement was not significantly different between the different conditions for either direction of perturbation (*P* > 0.05). For anterior perturbations, peak CoP displacements were slightly larger with eyes closed (mean EO 47.9 mm, mean EC 56.8 mm; *P* = 0.03) but showed no difference in peak latency. There was no significant difference in baseline EMG levels for any muscle group between the different conditions tested (*P* > 0.05), but the SOL muscle group had slightly earlier onset latencies during eyes closed (mean EO 129.5 ms, mean EC 104.4 ms, *P* = 0.044). There were no effects of the conditions on EMG onset latency for TA, HS or PS (*P* > 0.05 for all). Anterior perturbations showed a larger increase in EMG amplitude for SOL over the initial 0.2 s during eyes closed (mean EO 33.9 µV, mean EC 38.2 µV, *P* = 0.014), but neither HS nor PS showed any difference across surface and vision types (*P* > 0.05).

For posterior perturbations, peak CoP displacements showed no significant difference between conditions. Peak CoP latencies were shorter for the compliant surface (mean rigid 667.6 ms, mean compliant 560.9 ms, *P* = 0.006) as were mid-return latencies (main effect of surface; *P* = 0.026). There was no significant effect of conditions on EMG onset latencies for TA, SOL or QUAD muscle groups (*P* > 0.05 for all), but EMG amplitudes for TA were larger for the compliant surface from the onset of the perturbation (0 s) to 0.75 s afterwards (interaction between surface and time interval; *P* = 0.013). Neither QUAD nor SOL EMG amplitudes were significantly different across the surface and vision types (*P* > 0.05). Given the limited differences, the results for all four conditions were averaged for each direction of perturbation (Supplementary Figure).

## Discussion

The responses shown in this study differ substantially from those we previously reported for taps applied at a similar truncal level (Govender et al. 2015). Although the peak accelerations used here were no bigger than in our previous report, they were sustained for longer and, given displacement scales as the square of the duration, implied a greater displacement at C7. The body did not behave like a rigid object; rather the acceleration wave propagated down the axial structures and was both delayed and attenuated by the time it reached the sacrum. Unlike the briefer stimuli we used previously, the present ones represented a threat to postural stability and required specific intervention. The initial responses did not simply consist of excitation of an agonist and inhibition of its antagonist. While the grand average did show a very short period of inhibition prior to the main response of soleus for posterior perturbations, consistent with the reflex effect we reported previously, this was largely replaced by a short period of cocontraction (Fig. 3, Supplementary Fig.). It would appear that when a disturbance becomes too large for simple reflexes to compensate for, more complex responses are recruited. The initial phasic response was followed by a second, prolonged contraction, with reciprocal activation of soleus and TA. Our subjects had initial trials to acquaint them with the disturbance so can be assumed to have adopted an appropriate response consisting of both automatic and voluntary components.

The response to the pull-forward displacement was the more straightforward and was similar to those reported for platform displacements posteriorly. In both cases, the body’s centre of mass is displaced forwards and the response mainly consists of a contraction of the dorsal muscles—the soleus, the hamstrings and the paraspinal muscles, to counteract the applied force (Horak and Nashner 1986; Horak et al. 1994). We did not demonstrate an earlier latency for soleus than for more proximal muscles, and the pattern of recruitment was consistent with near simultaneous, short latency activation of all three muscle groups. This may be due to the smaller role of ankle proprioceptors in the response and as well as the contributions from more proximal receptors. Horak et al. (1994) also found near simultaneous, shorter latencies for responses to perturbations applied to the head and which they presumed were mediated through vestibular afferents.

The response to posterior perturbations differed substantially from those shown for corresponding platform displacements. It has been demonstrated that two distinct patterns of postural response, the “ankle” and “hip” strategies, are possible means to compensate for body displacements induced by a moving platform. Subjects can switch their response set to adopt the “hip” strategy in which the main muscle activation occurs around in hip muscles, when standing on a short base (Horak and Nashner 1986). The biomechanical considerations for backwards truncal displacements are significantly different from anterior ones. For forward displacements on a firm surface, the calf muscle contraction is resisted by an equal sized reactive force from the surface which can in turn be transmitted through the contracting dorsal muscles. In contrast, for posterior disturbances, the anterior shank muscles, which dorsiflex the foot, have no such resistive force, so the maximum force acting to restore the trunk anteriorly that can be achieved by contraction of the ankle dorsiflexor muscles is limited and depends upon the posture of the trunk. If the body were rigid, the maximum restoring acceleration that could be achieved when contracting the dorsiflexors of the ankles can be no more than gravity times the cosine of the angle of the trunk to the horizontal. Thus, only by adopting a posture with a forward lean is a (limited) restoring force available. Thus, simply contracting ventral muscles can only overcome a weak posterior perturbation. A more complex response set than simply contracting ventral muscles was adopted in our experiments, demonstrated by the initial paradoxical forwards acceleration measured at the two tibias. Subjects presumably chose the more complex response set following their initial exposure to the stimulus. There was significant activation of the TAs and quadriceps muscles but no activation of the abdominal muscles. Subjects appeared to be absorbing some of the applied force to rotate the trunk around the centre of mass, with the knees going forwards and transiently adopting a posture like that used to “limbo”, before straightening up. This can be expected to absorb a larger force than would be possible using a uniform ventral muscle contraction, given the physical limitation on the amount of force that can be generated to accelerate the trunk anteriorly.

Although the CoP displacement onset was significantly earlier in response to imposed perturbations than for voluntary movement, this might have simply been due to the initial effects of the imposed perturbation itself. Overall, the CoP recordings under our conditions will be dominated by changes in local force production rather than movement of the centre of gravity. Calculations based on the sacral accelerometer recordings suggest the displacement of the centre of gravity was less than 20 mm. For anterior perturbations, there was no significant latency difference between the EMG onsets from the corresponding voluntary movement (Fig. 5), whereas there was a significantly shorter onset for TA during posterior perturbations. For posterior perturbations, an initial cocontraction was present but not for voluntary movement; indeed in some individuals, we found that the initial contraction was counter-compensatory (e.g. initial TA contraction with anterior perturbations). The distinction between “reflex”, “automatic” and “voluntary” movements is not easily defined (e.g. Prochazka et al. 2000), but the initial part of the postural response was different from the voluntary response to a tap stimulus. The differences included two phases of the response, with the tendency for cocontraction for the initial phasic discharge and, for posterior displacements, the latency of the onset of the response. The term “automatic” appears to be appropriate to describe the responses that we saw, but the earliest contractions appear not to have been generated by the normal cortical mechanisms underlying voluntary movement. The effect is unlikely to be mediated by long-latency stretch reflexes as there was excitation of both agonist and antagonist muscles. Brown et al. (1991) showed that standing shortened the onset of the startle reflex and reduced the median latency in TA to just over 80 ms, similar to the latencies we observed for the posterior perturbations. Cocontraction would act to increase muscle stiffness around the joints, but the relative levels of activity in TA and soleus during the period of cocontraction varied for the two directions of perturbation, suggesting some specificity for even this response (Fig. 3). Following the initial period of cocontraction, there was a sustained increase in TA activity (for posterior perturbations) that was greater than that obtained for the voluntary displacement. It may be that reticulospinal pathways contributed both to the initial phasic activation and possibly also to that following (Valls-Solé et al. 2008; Shemmell 2015), the latter occurring in parallel with corticospinal outflow.

The effects of different stance conditions indicated that vision made little contribution to the responses. Given the delay in the acceleration wave transmitting through the skeleton, it is likely that proprioceptive afferents at the ankles would have been activated too late to contribute to the initial response to the applied perturbations. The compliant surface, a condition under which proprioceptive and cutaneous information from the ankles and feet would be less reliable, was associated with an enhancement of the postural response. Vestibular afferents could have contributed, and indeed vestibular reflexes are enhanced with compliant surfaces (Welgampola and Colebatch 2001). Study of postural responses to upper trunk perturbations in patients with vestibular impairment will be important.. However, we and others have previously reported that vestibular afferent information is less potent than afferents apparently arising from truncal receptors, evoked by similar levels of acceleration (Govender et al. 2015), and it is likely that these truncal afferents also provide important inputs to trigger the postural responses that we have shown.

## Electronic supplementary material

Supplementary material 1 (PDF 406 kb)

Supplementary material 2 (DOCX 16 kb)

Supplementary material 3 (DOCX 15 kb)
