# Supplementary material for: Postural responses to anterior and posterior perturbations applied to the upper trunk of standing human subjects
Source: Exp Brain Res. 2015 Oct 20;234:367–76. doi: 10.1007/s00221-015-4442-2 (PMC4731437; doi:10.1007/s00221-015-4442-2)
Supplement: Supplementary file 1 — Supplementary material 1 (PDF 406 kb) [file 221_2015_4442_MOESM1_ESM.pdf]

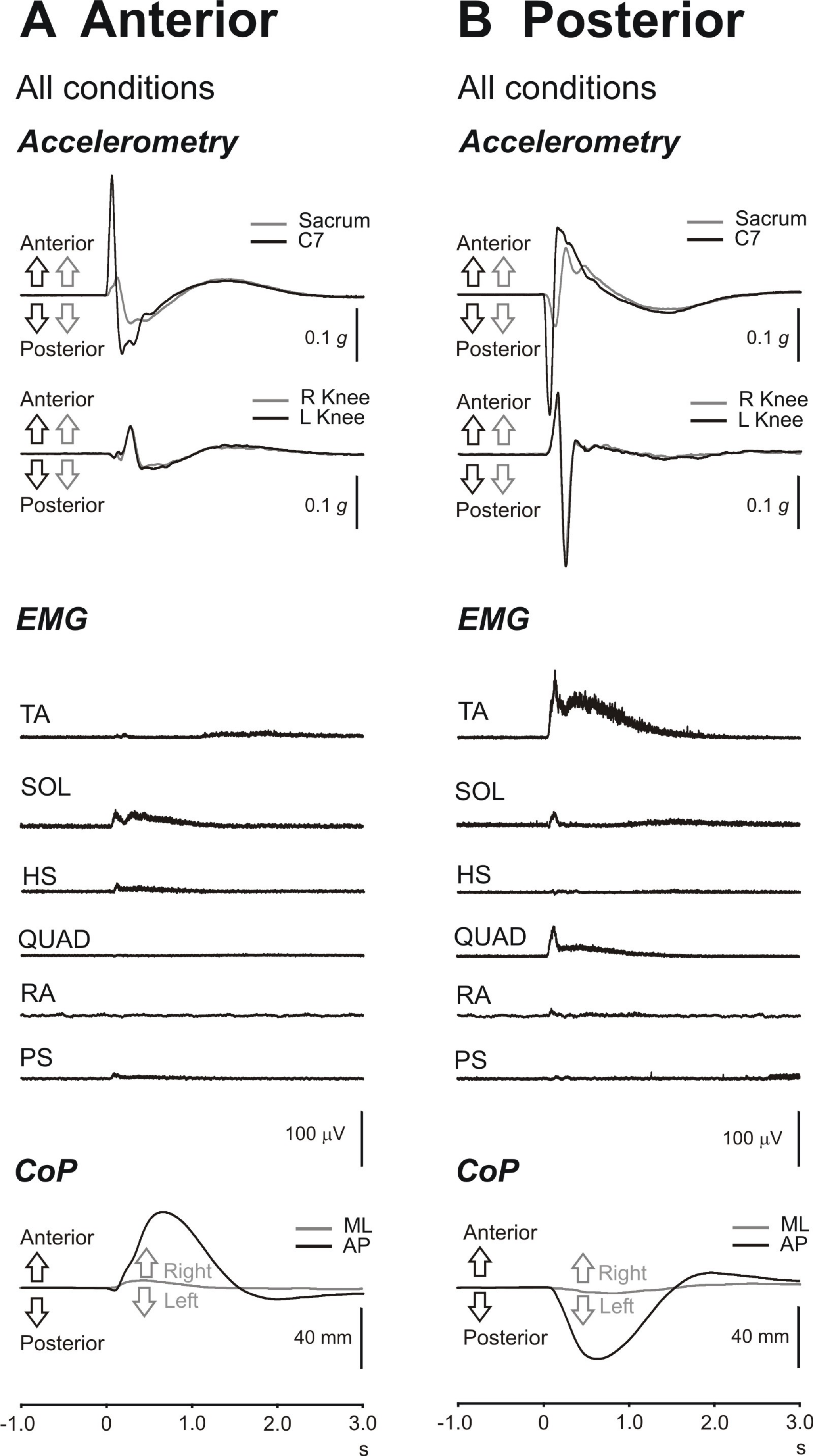
A Anterior
All conditions
Accelerometry
Anterior
Posterior
Sacrum
C7
0.1 g
R Knee
L Knee
EMG
TA
SOL
HS
QUAD
RA
PS
100 μV
CoP
ML
AP
Right
Left
40 mm
-1.0
0
1.0
2.0
3.0
s
B Posterior
All conditions
Accelerometry
Anterior
Posterior
Sacrum
C7
0.1 g
R Knee
L Knee
EMG
TA
SOL
HS
QUAD
RA
PS
100 μV
CoP
ML
AP
Right
Left
40 mm
-1.0
0
1.0
2.0
3.0
s
