# Supplementary material for: Postural responses to anterior and posterior perturbations applied to the upper trunk of standing human subjects
Source: Exp Brain Res. 2015 Oct 20;234:367–76. doi: 10.1007/s00221-015-4442-2 (PMC4731437; doi:10.1007/s00221-015-4442-2)
Supplement: Supplementary file 2 — Supplementary material 2 (DOCX 16 kb) [file 221_2015_4442_MOESM2_ESM.docx]

**Supplementary Table 1: Acceleration, CoP and EMG values for voluntary anterior lean conditions**

|  | **Rigid surface** | |  | **Compliant surface** | | | |
| --- | --- | --- | --- | --- | --- | --- | --- |
|  | **EC** | |  | **EO** | | **EC** | |
| **Acceleration** | Peak Amp.  (m*g*) | Peak Lat.  (ms) |  | Peak Amp.  (m*g*) | Peak Lat.  (ms) | Peak Amp.  (m*g*) | Peak Lat.  (ms) |
| C7 | 26.6 (7.4) | 300.6 (96.5) |  | 24.8 (8.3) | 286.3 (68.1) | 19.1 (7.7) | 319.5 (67.0) |
| Sacrum | 21.2 (16.9) | 358.7 (42.6) |  | 16.2 (10.0) | 367.7 (71.0) | 15.8 (10.6) | 365.4 (37.4) |
| **CoP** | Displacement (mm) | Latency  (ms) |  | Displacement (mm) | Latency  (ms) | Displacement (mm) | Latency  (ms) |
| AP peak  displacement | 66.9 (40.7) | 1085.8 (234.8) |  | 69.4 (33.4) | 1211.6 (238.2) | 63.3 (33.7) | 1244.5 (292.6) |
| Onset | - | 120.9 (11.3) |  | - | 116.3 (9.4) | - | 125.3 (6.0) |
| Mid return | - | 1568.8 (350.6) |  | - | 1690.0 (357.3) | - | 1736.7 (366.6) |
| **EMG** | Median Lat.  (ms) | |  | Median Lat.  (ms) | | Median Lat.  (ms) | |
| TA | 113.9 [109.1-218.3] | |  | 111.5 [101.1-132.9] | | 135.1 [111.3-157.8] | |
| SOL | 180.2 [112.5-251.0] | |  | 137.8 [130.8-200.8] | | 176.1 [129.1-207.0] | |

**EC = eyes closed, EO = eyes open, Amp. = amplitude, Lat. = latency. Values are given as mean (SD) for acceleration and CoP. Onset EMG latencies are given as median [range]. Positive and negative values (for acceleration and CoP measurements) indicate anterior and posterior directions respectively.**
