# Supplementary material for: Postural responses to anterior and posterior perturbations applied to the upper trunk of standing human subjects
Source: Exp Brain Res. 2015 Oct 20;234:367–76. doi: 10.1007/s00221-015-4442-2 (PMC4731437; doi:10.1007/s00221-015-4442-2)
Supplement: Supplementary file 3 — Supplementary material 3 (DOCX 15 kb) [file 221_2015_4442_MOESM3_ESM.docx]

**Supplementary Table 2: Acceleration, CoP and EMG values for voluntary posterior lean conditions**

|  | **Rigid surface** | |  | **Compliant surface** | | | |
| --- | --- | --- | --- | --- | --- | --- | --- |
|  | **EC** | |  | **EO** | | **EC** | |
| **Acceleration** | Peak Amp.  (m*g*) | Peak Lat.  (ms) |  | Peak Amp.  (m*g*) | Peak Lat.  (ms) | Peak Amp.  (m*g*) | Peak Lat.  (ms) |
| C7 | -28.9 (14.1) | 302.3 (55.2) |  | -23.9 (8.2) | 287.0 (71.4) | -20.7 (9.0) | 289.1 (69.1) |
| Sacrum | -28.7 (25.7) | 285.6 (78.0) |  | -19.5 (10.3) | 256.2 (50.0) | -11.6 (5.7) | 279.6 (78.5) |
| **CoP** | Displacement (mm) | Latency  (ms) |  | Displacement (mm) | Latency  (ms) | Displacement (mm) | Latency  (ms) |
| AP peak  displacement | -53.4 (21.6) | 978.7 (166.8) |  | -49.9 (20.8) | 1104.6 (124.4) | -53.5 (21.3) | 1107.1 (181.6) |
| Onset | - | 124.2 (24.8) |  | - | 114.4 (19.1) | - | 130.0 (21.3) |
| Mid return | - | 1511.5 (279.8) |  | - | 1684.1 (322.3) | - | 1727.0 (250.6) |
| **EMG** | Median Lat.  (ms) | |  | Median Lat.  (ms) | | Median Lat.  (ms) | |
| TA | 157.9 [101.0-249.4] | |  | 179.5 [116.1-282.5] | | 179.2 [133.8-288.0] | |
| SOL | 121.3 [99.6-194.9] | |  | 122.2 [104.1-122.6] | | 147.3 [108.6-224.9] | |

**EC = eyes closed, EO = eyes open, Amp. = amplitude, Lat. = latency. Values are given as mean (SD) for acceleration and CoP. Onset EMG latencies are given as Median [range]. Positive and negative values (for acceleration and CoP measurements) indicate anterior and posterior directions respectively.**
